# Does motivational interviewing improve the weight management process in adolescents? Protocol for a systematic review and meta-analysis

**DOI:** 10.1186/s13643-018-0814-6

**Published:** 2018-10-30

**Authors:** Parisa Amiri, Elham Kazemian, Mohammad Masih Mansouri-Tehrani, Ahmad Khalili, Atieh Amouzegar

**Affiliations:** 1 Research Center of Social Determinants of Health, Research Institute for Endocrine Sciences, Shahid Beheshti University of Medical Sciences, Tehran, Iran; 2 Endocrine Research Center, Research Institute for Endocrine Sciences, Shahid Beheshti University of Medical Sciences, P.O. Box: 19395-4763, Tehran, Iran; 3 Department of Basic Sciences and Cellular and Molecular Nutrition, Faculty of Nutrition Sciences and Food Technology and National Nutrition and Food Technology Research Institute, Shahid Beheshti University of Medical Sciences, Tehran, Iran; 4 Student Research Committee, School of Medicine, Shahid Beheshti University of Medical Sciences, Tehran, Iran

**Keywords:** Motivational interviewing, Obesity, Adolescent

## Abstract

**Background:**

Data on the effects of motivational interviewing (MI) on modifying unhealthy lifestyles and promoting weight status during childhood is controversial. Adolescents are more prone to assume higher personal responsibilities for behavioral changes. This study aims to investigate whether MI will improve weight management process in adolescents.

**Methods/design:**

A systematic review will be conducted on clinical trials, assessing the effect of MI on weight management processes in adolescents, aged 10 to 19 years. The primary objective is to assess the efficacy of MI in controlling weight-related measures in overweight and obese adolescents. Secondary objectives are assessing the efficacy of MI on obesity-related behaviors and cognitive abilities considering heterogeneity in outcomes of primary studies in different MI settings. Main data sources include MEDLINE/PubMed, Scopus, Embase, Web of Science, Cochrane, and PsycINFO from 1980 to May, 2018, with no language restrictions. Study selection, data extraction, and risk of bias assessment will be performed by two independent reviewers. A meta-analysis will be conducted on relevant outcomes. Data will be analyzed for outcome of interest using the 95% confidence interval (CI) of an estimate for dichotomous outcomes and mean differences (MDs) for continious outcomes. Cochrane’s *Q* statistic and the *I*^2^ statistic will be performed to evaluate the heterogeneity. Subgroup analysis and suitable analytical strategies will be conducted to identify the potential sources of heterogeneity. As we expect a high heterogeneity in our included studies, pooled risk ratios (RRs) and 95% CI will be calculated to estimate the overall effect sizes, using meta-regression models or finite mixture modeling through conducting random effect methods. GRADE system will be used to evaluate the certainty of evidence. We will also use subgroup analysis and the GRADE system to investigate the effect of methodological quality of primary studies on results of meta-analysis. Funnel plots and egger and beggs test and plot will be implemented to assess publication bias.

**Discussion:**

The results of this systematic review will provide more insights regarding the effect of MI on weight management in adolescents and will be useful for future research and health promotion programs in this age group.

**Systematic review registration:**

PROSPERO 2017:CRD42017069813.

## Background

Childhood obesity has become a major problem in the twenty-first century [1] in both developing and developed countries [2] raising much concern among health care providers. The International Obesity Task Force (IOTF) reported that one in every ten children worldwide is overweight [3]. Excessive weight gain among children can adversely affect both their physical and emotional health [4] and it can not only result in adulthood obesity, but it can also lead to increased risk of many related chronic diseases including diabetes type 2 (44%), ischemic heart disorders (23%), and certain cancers (7–41%) throughout life, causing at least 2.8 million deaths annually [5, 6].

Behavioral interventions to modify both diet and daily physical activity have been demonstrated to be the most effective strategy for weight management in children and adolescents [7–10]. In this regard, several cognitive behavioral strategies, specifically those focused on the stimulus control and lifestyle self-monitoring, have been shown to be able to support healthy weight in childhood [10–14]. As a patient-centered counseling style, “MI is a collaborative, person-centered form of guiding to elicit and strengthen motivation for change” [15], designed to strengthen personal commitment by respecting individuals’ autonomy and help them to reach a specific goal by exploring their own reasons [16]. Motivational interviewing (MI) includes two essential components: (1) the relational component known as the MI spirit, which emphasizes on showing empathy, eliciting patients thoughts, respecting the patient’s autonomy, using open-ended questions, and appreciating patient’s reflections; (2) the technical component which put emphasis on using open-ended questions and reflective listening to delineate patients’ arguments for change, known as “change talk” [16, 17]. MI is being widely used for triggering behavior change and it has been proposed to be effective in promoting healthy behaviors and enhancing weight loss in obese and overweight individuals [18–20].

As a transition period from childhood to adulthood, adolescence, which coincides with physical, metabolic and emotional development and the person’s increased independency, is a golden age to encourage healthy behaviors [21]. Adolescents are more prone to assume higher personal responsibilities for behavioral changes and choices [22]. As a patient-oriented communication style, MI expects the patient, as opposed to the health provider, to use his/her reasoning to promote their life style which leads to more collaboration and trust in both parties, resulting in higher levels of autonomy and patient satisfaction [21, 23].

Among previous systematic reviews which have specifically investigated the effectiveness of MI on weight management, adult populations have been mostly targeted [18, 24–27]. In addition, a number of systematic reviews have been conducted on lifestyle interventions, using MI as one of the behavioral strategies to manage excessive weight gain in children [6, 28–34]. To the best of our knowledge, only two reviews conducted by Resnicow et al. [34] and Borrello et al. [28] specifically addressed the effect of MI on childhood lifestyle modification and weight control; the first was a brief narrative review conducted on two primary studies of children, aged 3–18 years, which makes it difficult to interpret the results and reach a conclusion, and the second encompassed six original studies conducted on children 2–11 years old, but not adolescents. Despite the critical importance of adolescence as a period for lifestyle modification, there are no systematic reviews regarding the effect of MI on the weight management process in adolescents specifically. A better understanding of the effects of MI on health-related outcomes can provide the insight needed for health care professionals to make the best decisions for health-related policies. This review will be performed to estimate the effects of motivational interviewing on weight management processes in adolescents.

## Objectives

### Primary objectives

Evaluating the effects of motivational interviewing to control weight-related measures, i.e., body mass index (BMI), BMI *z*-score, waist circumference (WC), hip circumference (HC), waist to hip ratio (WHR), height to weight ratio (HWtR), in overweight and obese adolescents, aged 10–19 years.

### Secondary objectives

To assess:

1. The effect of motivational interviewing on obesity-related behaviors, including physical activity (PA) and dietary patterns to manage obesity.
2. The effect of motivational interviewing on cognitive abilities, including self-efficacy, self-regulation, and self-control to manage obesity.
3. Heterogeneity in primary studies regarding methodological factors (e.g., different settings, MI mode of delivery, etc.) which could influence the effect of motivational interviewing on weight management process.

## Methods/design

The present systematic review/meta-analysis protocol will be conducted in adherence to the checklist guidelines recommended by Preferred Reporting Items for Systematic Review and Meta-Analysis (PRISMA) and Cochrane [35–37]. This protocol has been registered with the PROSPERO international prospective register of systematic reviews, under the identification code: PROSPERO 2017: CRD42017069813.

### Selection criteria

We conducted a pilot test on ten articles to determine the inclusion and exclusion criteria before the screening phase.

#### Type of studies

All randomized controlled trials (RCTs) will be screened for recruitment in the current review based on the main objectives, using the participants, interventions, comparisons, outcomes (PICO) criteria. Studies including review articles, all observational studies, case studies and case reports, commentaries, and opinion papers will be excluded. No language restrictions will be applied to trial eligibility. In this regard, the article (s) will be translated to English (by native speakers or translating web-sites), and if they meet our inclusion criteria, they will be included in our study. Narrative and systematic reviews will be excluded but those relevant to our objective of interest for discussion and additional references will be scanned.

#### Type of participants

Primary studies will be included if they have been conducted on adolescents (boys and girls, aged 10–19) without any physical and mental diseases (type II diabetes patients, alcohol dependence, hypertension, cancers, depression, serious mental illness, musculoskeletal disorders, nonalcoholic fatty liver disease, cardiovascular disease, inflammatory bowel disease, perinatal situation, chronic disease, metabolic syndrome, eating disorders, etc.) and those not receiving any other care. We will include studies having MI as a treatment intervention to improve the weight management process in adolescents.

#### Intervention and control

We will include all primary studies which have explicitly used MI as an approach to motivate adolescents for behavioral modification and weight management in comparison to control group. In this regard, based on the results of our preliminary search, the minimum follow-up time for the selected studies will be 3 months.

Control groups will receive either no intervention or receive other behavioral (PA/nutritional counseling) interventions to control weight, but will not be receiving any motivation-based interventions.

#### Outcomes

Anthropometric measures including BMI, BMI *z*-score, WC, HC, and WHR, as indicators for managing obesity, will be regarded as primary outcomes. Our secondary outcomes will be the effectiveness of MI in daily PA, dietary patterns, adherence to nutritional counseling and PA programs, as well as cognitive abilities including self-efficacy, self-regulation, and self-control; we will also investigate adolescents’ weight maintenance within normal range, based on their age and sex, after completion of intervention period. Primary studies will be included if they report one of anthropometric measures of interest, irrespective of other primary or secondary outcomes.

### Search strategy

A comprehensive review of literature available will be conducted by searching the following electronic databases from 1980/01/01 until May 2018, with no language restriction.

#### General databases

Medline via PubMed, Elsevier via Scopus, Elsevier via Embase, ISI via web of science, and Cochrane Central Register of Controlled Trials (Clinical Trials).

#### Database of expertise

American Psychological Association, PsycINFO (http://www.apa.org/pubs/databases/psycinfo/).

#### Key journals

BMC public health, Contemporary clinical trials, Patient education and counseling, Childhood obesity, International journal of Obesity, JAMA pediatrics, Pediatric obesity, Pediatrics.

#### Other sources

References of the relevant papers

Search terms include “overweight” or “weight loss” or “obese” or “obesity” or adiposit* or “fat overload” or “adipose tissue hyperplasia” and “motivat* interview*” or “motivat* counsel*” or “motivat* therapy” or “motivat* therapies.”

Our systematic review will be conducted on adolescents, aged 10–19 years. In our search syntax, the term “adolescent” was not used, since we want to include all studies with other age groups that may be involved in our eligible age group.

Relevant search terms in accordance with search components (intervention and outcome components of current systematic reviews) were identified from MeSH, EMTREE terms, and throughout reviewing abstracts of related articles. Furthermore, bibliographies of all related previous systematic reviews and meta-analyses and primary studies found by search strategies will be scrutinized for additional relevant papers. Gray literature including conference proceedings will be identified by searching Scopus and Web of Science and annual meetings documented. In case of included papers in which the required data are not provided, corresponding authors will be contacted to ask for the data. As the first step, the syntax in PubMed will be finalized and then it will be modified and used for other databases. Search results will be imported into endnote, and then key journals and other sources will be scanned for potential relevant articles. Duplicate references will be deleted based on the author, year, and title. In the case of gray literature, if their original paper exists, the full text will be read to determine the eligibility for inclusion. Search syntaxes in six main data bases are presented in Table 1.

**Table 1:** Search syntax for the databases

Databases	Final syntax	Description	Records number
PubMed	((Obesity[tiab] OR “”[tiab] OR “weight loss”[tiab] OR obese[tiab] OR adiposit*[tiab] OR “fat overload”[tiab] OR “adipose tissue hyperplasia”[tiab]) AND ((motivat*[tiab]) AND (interview*[tiab] OR therapy[tiab] OR therapies[tiab] OR counsel*[tiab]))) AND 1980/01/01:2018/05/06[dp]	Journal papers	955
Scopus	((TITLE-ABS(Obesity) OR TITLE-ABS(“overweight”) OR TITLE-ABS(“weight loss”) OR TITLE-ABS(obese) OR TITLE-ABS(adiposit*) OR TITLE-ABS(“fat overload”) OR TITLE-ABS(“adipose tissue hyperplasia”)) AND ((TITLE-ABS(motivat*)) AND (TITLE-ABS(interview*) OR TITLE-ABS(therapy) OR TITLE-ABS(therapies) OR TITLE-ABS(counsel*)))) AND (PUBYEAR > 1980)	Journal papers	937
		Conference papers	33
		Others	49
Embase	((Obesity:ti,ab OR “overweight”:ti,ab OR “weight loss”:ti,ab OR obese:ti,ab OR adiposit*:ti,ab OR “fat overload”:ti,ab OR “adipose tissue hyperplasia”:ti,ab) AND ((motivat*:ti,ab) AND (interview*:ti,ab OR therapy:ti,ab OR therapies:ti,ab OR counsel*:ti,ab))) AND [1980–2019]/PY	Journal papers	839
		Conference papers	507
		Others	30
WOS	((TS = (Obesity) OR TS = (“overweight”) OR TS = (“weight loss”) OR TS = (obese) OR TS = (adiposit*) OR TS = (“fat overload”) OR TS = (“adipose tissue hyperplasia”)) AND ((TS = (motivat*)) AND (TS = (interview*) OR TS = (therapy) OR TS = (therapies) OR TS = (counsel*)))) AND PY = (1980–2019)	Only journal papers	1057
		Meeting abstract	20
		Proceeding paper	39
		Others	14
Cochrane	(Obesity OR “overweight” OR “weight loss” OR obese OR adiposit* OR “fat overload” OR “adipose tissue hyperplasia”) in Title, Abstract, Keywords AND ((motivat*) AND (interview* OR therapy OR therapies OR counsel*)) in Title, Abstract, Keywords, Publication Year from 1980 to 2018 in Trials’	Only journal papers(trials)	528
PsycINFO	((Obesity or “overweight” or “weight loss” or obese or adiposit* or “fat overload” or “adipose tissue hyperplasia”) and (motivat* and (interview* or therapy or therapies or counsel*))).mp. [mp = title, abstract, heading word, table of contents, key concepts, original title, tests & measures]	All papers	549
Total			5557
Duplicate			2267
Total after deleting duplicates(remaining for screening)			3290

### Data collection and analysis

#### Study selection

Studies will be included based on Preferred Reporting Items for Systematic Reviews and Meta-Analyses (PRISMA) flow diagram [36]. Studies will be screened based on titles and abstracts by two reviewers (MMMT and AK) using a standardized eligibility tool, including inclusion and exclusion criteria. Endnote will be used to manage screening and selecting stage. First, the titles and abstracts of all primary studies will be scanned by two reviewers to ascertain adequacy of studies for inclusion; then, we will obtain the full text of all the remaining potentially relevant articles, which will be assessed against the eligibility criteria by two independent reviewer authors (PA and EK) for final inclusion. In this regard, reviewers will be given copies of all full texts of studies screened and will be asked to complete tables which include three items (include, exclude, and border line of suspicious studies) based on inclusion and exclusion criteria independently. We will evaluate eligibility criteria for each study in order of importance. The predetermined hierarchy of reasons for excluding the ineligible studies on the basis of importance will be as follows: different intervention (not MI), different age range, different aim and design, different study groups (those with normal weight or with chronic diseases), and lack of reports on eligible primary outcomes. Only one failed eligibility criterion will be sufficient for a study to be excluded from the review; therefore, the first “rejected” response will be the main reason for exclusion of the study, and the remaining will not be assessed.

#### Quality assessment

The methodological quality of included studies will be independently appraised by PA and AK using an adapted Cochrane Collaboration’s tool for assessing risk of bias in randomized trials before any retrieval of information (Table 2) [38]. Each study will be rated and allocated to one of the three following categories (1) high risk of bias, (2) low risk of bias, and (3) unknown risk of bias. We will not exclude trials with high risk of bias.

**Table 2:**
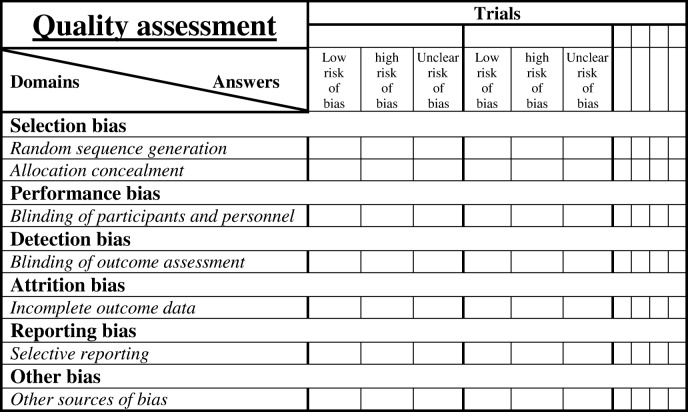
Adapted Cochrane collaboration’s tool for assessing risk of bias

#### Data extraction

Data extraction form will be designed and piloted, following which two independent reviewers (MMMT, AK) will be assigned to extract data from primary articles, using a quantitative data extraction form, which encompasses:

1. Study characteristics: Title of article, journal title, format (summary, journal article, gray literature), first author’s name, year of publication, country in which the study was performed, study design, sample size, and duration of follow-up.
2. Participants’ characteristics: Age, gender, category of obesity (obese or overweight), secondary disease, other primary or secondary care, study setting, and patient enrollment strategies and ethnicity.
3. Intervention and comparator details: Sample size for each treatment group, blinding, frequency and duration of the motivational program, other strategies for weight management as a co-intervention; mode of delivery (directly face to face or indirectly by phone).
4. Outcome measures: Any measurement of the current primary outcomes (BMI, BMI *z*-score, WC, HC, WHR) and secondary outcomes (changing of daily PA and energy expenditure, changing of nutritional behaviors and energy intake, adherence to nutritional counseling and PA programs, improving cognitive abilities including self-efficacy, self-regulation and self-control compliance, lifestyle modification maintenance, and heterogeneity).
5. Key measures for meta-analysis, including relative risk, mean difference, standard mean difference, standard deviation, standard error, odds ratio, mean, and sample size.

With regards to duplicated studies, author will be contacted to make sure whether the studies are duplicates, and the stronger study will be included. For example between a conference paper and a journal article, the latter one will be included as the most explicit document.

#### Dealing with missing data

We will also try to contact authors of the studies with possibly relevant but unpublished data; if no response is received from the author(s) of such publications, they will be contacted three times at intervals of 15 days. In cases where no responses are received, we will calculate the missing data from other measures or estimate them from the most similar study; finally, if we cannot extract these data, we will exclude them.

#### Dealing with disagreement

To assess the inter-rate agreement between reviewers through different phases of study including study selection, data extraction, and quality assessment, the kappa index will be computed [39] and any disagreement arising between the two reviewers will be resolved via discussion or referral to a third reviewer (if necessary) to reach consensus. We will record the reasons for excluding each paper at any of the above mentioned stages.

#### Descriptive analysis

For all studies included, a table of descriptive characteristics will be provided, which will contain data on study characteristics, population, sample size for each treatment group, ethnicity, sex, age at baseline, category of obesity, social background, duration and frequency of the motivational program, mode of delivery, co-intervention, and outcomes. We anticipate that effectiveness or acceptability of the interventions of interest may be affected by some of the clinical and methodological variables. The clinical and methodological comparability of interventions will be assessed to ensure transmissibility after data extraction. We assume that there will be enough clinical trials to conduct a meta-analysis for the outcomes of interest.

#### Inferential statistics

We will present categorical and continuous variables as count or proportions and mean or medians respectively. We will synthetize data for outcome of interest by using the 95% confidence interval [19] of an estimate (for example, of odds ratios or relative risks) for dichotomous outcomes and mean differences (MDs) for continuous measures. We will calculate MDs by subtracting the mean change in the control group from that in the intervention group. Standard deviations (SDs) will be calculated from standard errors (SEs), or confidence interval (95% CI) for both the control and intervention groups. A between-group difference and relative differences in outcome will be calculated. All data manipulation and analyses will be carried out by using STATA (Stata Corp. 2013. Stata Statistical Software: Release 13. College Station, TX: Stata Corp LP).

#### Assessment of heterogeneity

Cochrane’s *Q* statistic (*p* value of < 0.05 will be considered statistically significant) and the *I*^2^ statistic will be implemented to examine the extent of heterogeneity in the studies; the *I*^2^ statistic will be categorized as 0–25% unimportant heterogeneity, 25–50% as moderate, 50–75% notable, and > 75% as considerable heterogeneity [40]. Any amount of heterogeneity is acceptable for meta-analysis, and the most appropriate methods, will be used for analysis of these heterogeneous studies [41].

#### Assessment of reporting bias

Funnel plots and Egger’s and Begg’s test and plot will be implemented to assess publication bias, and if there is a high degree of publication bias, we will use the Trim and Fill method to modify the findings.

### Data synthesis and sensitivity analysis

Since MI is a complex intervention and a moderate to high heterogeneity is anticipated [15, 42], we will apply sub-group analysis and suitable analytical strategies to control the effect of this probable heterogeneity [43, 44]. In this regard, pooled risk ratios (RRs) and 95% CI will be calculated to estimate the overall effect sizes using meta-regression models or finite mixture modeling through conducting random effect methods.

#### Assessing confidence

To assess the certainty of evidence through each outcome, the Grading of Recommendations Assessment, Development and Evaluation approach (GRADE) system and additional necessary guidance will be used [45–47]. The outcomes for the current study will be listed based on the methods, described in the Cochrane Handbook for Systematic Reviews of Interventions and will be presented in a “Summary of findings” table [35].

#### Trial quality

To present meta-analysis stratified according to the risk of bias, the subgroup analysis will be carried out to investigate interaction between unknown and high risk versus low risk groups, but due to the lack of power of meta-regression to compare results from studies at high and low risk of bias, and since the lack of a significant difference should not be interpreted as implying the absence of bias, in the current review, risk of bias will also be reduced by using the GRADE system [38].

## Discussion

In the past three decades, overweight and obesity in children and adolescents have risen substantially in most high-income as well as low and middle income countries [48]. Economic growth, industrialization, mechanized transport, urbanization, increased sedentary lifestyles, and a nutritional transition to processed foods and high calorie diets have led to higher prevalence of overweight and obesity worldwide [7].

Obesity is associated with concomitant or increased risk of almost all chronic conditions, i.e., diabetes, metabolic syndrome, cardiovascular diseases, stroke, certain cancers, poor mental health, and osteoarthritis. Hence adequate personal and public health care seem necessary to promote healthier lifestyles and prevent overweight and obesity, particularly in vulnerable groups.

Despite the abundance of strategies for excessive weight management, applying a comprehensive, efficient, and commodious approach is a controversial topic. Several studies have assessed the effectiveness of motivational interviewing as a strategy for managing obesity and its related behaviors. While health-related habits of adults are affected by decisions which have been made in adolescence [49], to the best of our knowledge, none of the previous systematic reviews and meta-analyses have focused on the effect of motivational interviewing on excessive weight management in adolescents. Our systematic review will provide valuable information regarding the effect of MI on obesity-related behaviors and weight status in adolescents, which could help practitioners and health care providers to confirm whether using MI for weight management process is an appropriate strategy in this important group of individuals.

### Strengths and limitations of this study

This systematic review and meta-analysis, for the first time, will be conducted to evaluate the effectiveness of MI on the weight management process in adolescents according to the Preferred Reporting Items for Systematic Review and Meta-Analysis Protocols (PRISMA-P) recommendations using a comprehensive search of related databases with no language restrictions and including gray literature. Regarding the complexity of MI, maximum effort will be made to perform GRADE, using additional guidelines addressing how to use GRADE for evaluating the certainty of evidence [47]. Two reviewers will independently conduct full-text selecting, data extraction, and risk of bias assessment of the primary studies included. However, considering MI as a complex intervention, high heterogeneity in primary studies and outcomes would be expectable which may be intensified by a probable wide range of co-interventions. Last but not least, we may not find the qualified articles sufficient to perform a strong systematic review and meta-analysis.

### Trials status

The study was initiated on 31 July 2017 and will be completed on 31 October 2018.

## Abbreviations

(HWtR): Height to weight ratio
BMI: Body mass index
CI: Confidence intervals
HC: Hip circumference
MDs: Mean differences
MI: Motivational interviewing
PA: Physical activity
PICO: Participants, interventions, comparisons, outcomes
PRISMA: Preferred reporting items for systematic reviews and meta-analyses
RCTs: Randomized control trials
RRs: Risk rations
SEs: Standard errors
WC: Waist circumference
WHR: Waist to hip ratio
